# Toxicological Evaluation of *Camellia euphlebia* Leaves Aqueous Extract Using Acute and Subacute Toxicity Studies in Mice and Genotoxicity Studies

**DOI:** 10.1155/2022/7889199

**Published:** 2022-02-15

**Authors:** Dongye He, Yongping Xu

**Affiliations:** ^1^Department of Endocrinology, Genetics and Metabolism, Affiliated Hospital of Jining Medical University, Jining 272029, China; ^2^Medical Research Center, Affiliated Hospital of Jining Medical University, Jining 272029, China; ^3^School of Bioengineering, Dalian University of Technology, Dalian 116024, China

## Abstract

*Camelliaeuphlebia* is a novel food source and Chinese folk medicine with multiple pharmacological properties. Our previous exploration has demonstrated the antidepressant-like activity of *Camellia euphlebia* leaves aqueous extract by reliable animal models of depression; however, a lack of toxicological information limits its pharmacological application. The present study aimed to evaluate the preliminary safety of *C. euphlebia* extract by determining acute/subacute toxicity in mice and *in vivo*/*in vitro* genotoxicity. The oral-medium lethal dose of the extract in mice was found to be higher than 5000 mg/kg body weight in the acute toxicity study. In a 14-days subacute toxicity study, *C. euphlebia* extract at doses of 400, 800, and 1600 mg/kg did not result in significant changes in food intake, water intake, body weight, relative organ weight, aspartate aminotransferase activity, alanine aminotransferase activity, creatinine level, and number of white blood cells and red blood cells. However, histopathology observation of organs taken from all mice showed that 1600 mg/kg extract caused slight hydropic degeneration in the cytoplasm of hepatocytes. In a 28-days subacute toxicity study, 600 mg/kg extract significantly increased the level of red blood cells but produced no negative side effects on other pathological parameters. Mice treated with the extract at doses of 200, 400, and 600 mg/kg for 28 days did not manifest any histopathological alterations of the liver, kidney, and spleen. Additionally, the extract showed no chromosomal aberrations in the *in vivo* micronucleus test and *in vitro* chromosomal aberration test. The results revealed that the extract showed no significant toxic effects and no potential genotoxicity but with the likelihood of transient erythrocytosis and slight hepatotoxicity. Further chronic toxicological evaluation involved in more physiological parameters, especially associated with liver toxicity and erythropoietin level, would be needed to determine its safety and application value.

## 1. Introduction

Natural products derived from food or herbal medicine have been extensively used in the alternative treatment of ailments of mankind. For instance, antioxidants existed naturally in many beverages, fruits, vegetables, and medicinal plants have several health benefits in the prevention and treatment of many metabolic, cardiovascular, degenerative, and neoplastic diseases [1, 2]. Indeed, these nature-originated plant products do not actually exempt them from potential toxicity to humankind, albeit they are generally considered to be safer than chemically synthesized drugs [3]. Therefore, effective pharmacological activity and preclinical sufficient safety evidence are essential for the application actuality and foreground of natural products.

*Camellia euphlebia* Merr. ex Sealy (genus *Camellia*), praised as “flora panda,” is not only a rare ornamental species limitedly distributed in Southwest China and Northern Vietnam but also a novel food source authorized by the Chinese Ministry of Health [4, 5]. The great potential of *C. euphlebia*, either in respect of traditional folk medicine (urinary tract infection, dysentery, faucitis, and irregular menstruation) or modern pharmacological findings (antitumor, hypolipidemic, antidepressant, and antioxidant), has attracted considerable attention worldwide as a valuable resource for natural drugs [6]. Health-giving tea and tea-based drinks prepared from *C. euphlebia* flowers or leaves have been predominantly introduced into Southeast Asia, Europe, and American markets [7]. Although it has been applied to treat a wide range of ailments and development of functional products for a long time, toxicological profiles of *C. euphlebia* extract have not been scientifically investigated. Our study found that *C. euphlebia* aqueous extract (CEAE) performed antidepressant-like activity in multiple animal behavioral tests, such as forced swimming test (FST), tail suspension test (TST), open-field test (OFT), and chronic unpredictable mild stress test (CUMS), and its potential mechanism of action involved in the regulation of the unbalanced hypothalamic-pituitary-adrenal (HPA) axis and brain monoamine systems [8]. It was further elucidated that the antidepressant-like activity of CEAE was associated with neuroprotection by bidirectional regulation of the mitochondrial-mediated caspase-dependent apoptosis and PKA-pCREB signaling pathway in corticosterone-induced neuron injury model [9]. Hitherto, several classical antidepressants (TCAs, SSRIs, SNRIs, NaSSAs, SNaRIs, and NMDA receptor antagonist) that increase monoaminergic transmission within the synaptic cleft are extensively used in clinical prescription to treat the patients affected by depressive disorder. Nonetheless, these mainstream antidepressant agents caused various adverse effects, for instance, powerlessness, gastrointestinal disturbance, sleep-rhythm disorder, sexual dysfunction, headache, and vertigo [10, 11]. These adverse effects greatly limit the clinical application of current antidepressant agents, strongly reflecting the importance of safety evaluation in new drug discovery and development. As a prospective alternative treatment for depressive disorder, little toxicological data underpinned the necessity of preclinical safety testing of CEAE.

In the present study, we determined the biochemical, hematological, and histopathological changes after acute and subacute intragastric administration of CEAE in mice as well as genotoxicity potential by *in vivo* micronucleus test and *in vitro* chromosomal aberration test, with the aim to obtain preliminary safety information of *C. euphlebia*.

## 2. Materials and Methods

### 2.1. Plant Origin and Aqueous Extract Preparation

Fresh leaves of *C. euphlebia* were manually harvested from the Fangcheng city (Guangxi Zhuang Autonomous Region) and were authenticated by Prof. Zhonghhui Ma (College of Agriculture, Guangxi University, China), with a voucher specimen (8109255) preserved in digitized herbarium specimens in Guangxi Institute of Botany (China). Preparation of CEAE was performed using a procedure previously described [8]. Briefly, the hot air oven-dried leaves were processed by press-shear assisted interaction technology [12] and further extracted with an ultrasound-assisted aqueous extraction method under certain conditions (70°C, 40 KHz, 30 min). After centrifugation and filtration, the filtrate was freeze-dried and ultimately stored at −80°C until the next investigation. The main components in CEAE including total flavonoids, polysaccharides, polyphenols, saponins, catechin, quercetin, rutin, and caffeine have been detected by UV-spectrophotometry, high-performance liquid chromatography-mass spectrometry (HPLC-MS) or high-resolution mass spectrometry (HRMS) as previously reported [9].

### 2.2. Animals Grouping and Drug Administration

SPF female and male Kunming mice (23 ± 2 g) were purchased from the Experimental Animal Center of Dalian Medical University (Liaoning, China) and Jinan Pengyue Experimental Animal Co. Ltd. (Shandong, China). All mice were kept at appropriate relative humidity (50 ± 10%) and temperature (22 ± 2°C) under a 12 h light/dark cycle, with free access to both feed and water. They were acclimatized for at least 3 days and were randomly divided into different experimental groups (*n* = 10 per group, half male and half female) prior to testing. All procedures were carried out in strict accordance with the guideline of the Local Institutional Animal Care and National Act on the Care and Use of Laboratory Animals approved by Dalian Medical University (SYXK (Liao) 2008-0002, Dalian, China) and Jining Medical University (JNMC-2020-DW-FY-008, Jining, China).

**Figure 1:**
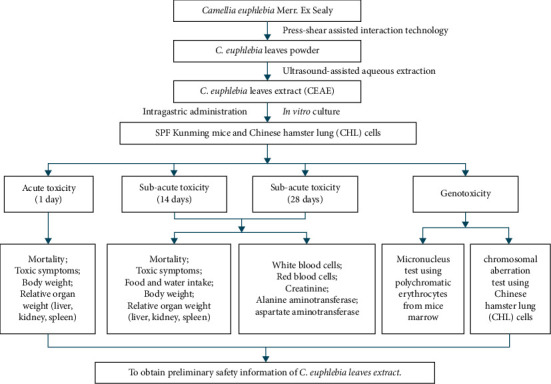
A detailed flowchart in text over the study design.

The animals were intragastrically administrated with saline and CEAE (0.4 mL/25 g body weight, BW) between 9:00 and 10:00 a.m., and each intragastric administration was carried out after a 12 h fasting period. For acute toxicity test, saline or CEAE was administered three times a day intermittently for 4 h, whereas saline or CEAE was given once per day in the 14-days or 28-days subacute toxicity test. Before each toxicity test, an appropriate dose gradient with toxic response was screened by preliminary experiment using wide dose ranges. A flowchart in text of the study design is shown in Figure 1.

### 2.3. Acute Toxicity Test

Mice in four experimental groups were administered with CEAE at doses of 1000, 2000, 4000, and 5000 mg/kg BW, respectively, while the control group only received the normal saline (Table 1). These doses of CEAE were 5–25 times higher than its antidepressant doses (200 mg/kg) [8]. The general behavior, toxic symptoms, and number of deaths per dose of mice in each group were recorded continuously for 1 h after intragastric treatment and thereafter over a period of 24 h, and further observed once a day up to 7 days [13]. The LD50 value was calculated according to the method of Litchfield and Wilcoxon [14]. All mice were weighed on day 1 (baseline) and day 7 (endline) followed by being sacrificed by cervical dislocation and further analysis of relative organ weight (liver, kidney, and spleen).

### 2.4. 14-Days Subacute Toxicity Test

The 14-days subacute toxicity study was carried out according to the OECD test guideline no. 425 [15]. Mice were randomly divided into one control group and three experimental groups and treated intragastrically with normal saline (0.4 ml/25 g) and CEAE (400, 800, and 1600 mg/kg) for 14 consecutive days. Toxic manifestations, mortality, food intake, water intake, and body weight of mice were monitored daily after drug administration. After all mice were sacrificed by cervical dislocation by the end of the experiment, creatinine, red blood cells, and white blood cells were analyzed by blood sample collected by cardiac puncture as well as relative organ weight (liver, kidney, and spleen) and the ALT and AST activities of the dissected liver tissue.

### 2.5. 28-Days Subacute Toxicity Test

The 28-days subacute toxicity study similar to the 14-days test was performed according to the OECD test guideline no. 407 [16]. Similar to the 14-days subacute toxicity study, mice were also randomly divided into one control group and three experimental groups but treated intragastrically with normal saline (0.4 ml per 25 g) and CEAE (200, 400, and 600 mg/kg) for 28 consecutive days. During the treatment period, general behavior, clinical signs of toxicity, mortality, food intake, water intake, and body weight of animals were recorded. At the end of the experiment (day 28), the blood sample was collected by cardiac puncture to analyze creatinine, AST, ALT, white blood cells, and red blood cells, whilst the liver, kidney, and spleen tissues were removed for analysis of relative organ weight.

### 2.6. Biochemical and Hematological Analysis

The blood sample was centrifuged at 4000 × g/4°C for 10 min to separate the serum, and 10% liver tissue homogenate diluted with normal saline was centrifuged at 2000 × g/4°C for 10 min to obtain the supernatant. Both the serum and liver supernatant were stored at −80°C for subsequent biochemical analysis. Creatinine, ALT, and AST in serum as well as ALT and AST in the liver tissue were determined by commercially available kits (C011-2/C010-2/C009-2, Nanjing Jiancheng Bioengineering Institute, Nanjing, China) according to the manufacturer's instructions. The hematological parameters including RBC and WBC were determined by commercially available kits (C037-1-1 and C025-1-1, Nanjing Jiancheng Bioengineering Institute, Nanjing, China) according to the manufacturer's instructions.

### 2.7. Histopathological Examinations of the Liver, Kidney, and Spleen Tissues

The liver, kidney, and spleen tissues of mice were immediately fixed in 10% phosphate-buffered formalin and were further embedded in paraffin blocks. Subsequently, these blocks containing tissues were then sliced into 6 *μ*m thick sections by a microtome and further stained with hematoxylin-eosin (H&E) solution (G1120, Solarbio, Beijing, China) for 5–10 min. The stained tissue section was observed with an inverted microscope (IX71 + DP71, Olympus, Japan) at 400× magnification to analyze histopathological changes in the liver, kidney, and spleen tissues.

### 2.8. Genotoxicity Studies

#### 2.8.1. In Vitro Chromosomal Aberration Test

The chromosomal aberration test was carried out using Chinese hamster lung (CHL) cells in line with the OECD test guideline no. 473 [17]. CHL cells were acquired from BeNa Culture Collection (Beijing, China) and cultured in the RPMI-1640 medium with 10% FBS, 100 ug/mL penicillin, and 100 ug/mL streptomycin (Gibco, CA, USA) at 37°C under the sterile condition of 5% CO_2_ and 95% air. Approximately 50% growth reduction is considered appropriate to determine the dose range of the tested substance [17]. Therefore, CEAE was finally set at 750, 1500, and 3000 ug/mL for 6 h treatment in the absence of S9 mixture (Moltox, NC, USA) and 300, 600, and 1200 ug/mL for 24 h treatment in the presence of S9 metabolic activation. Additionally, mitomycin C (MMC, 0.05 ug/mL) and cyclophosphamide (CPA, 10 ug/mL) (MedChemExpress, NJ, USA), both dissolved in water, were used as positive controls with and without S9 metabolic activation, respectively, whilst cells medium was served as the vehicle control. The CHL cells were seeded at a density of 1 × 10^5^ cells/well in 6-well microplates for 36 h. Subsequently, cells were treated with various doses of CEAE or positive/vehicle control drugs for a 6 h exposure followed by an 18 h recovery without S9 mixture (10%, v/v) as well as a 24 h exposure followed by 0 h recovery in the presence of S9 mixture. Four hours before the end of cell culture, colchicine (0.4 ug/mL) was applied to block cells in the metaphase. The cells were then collected, treated with 5 mL hypotonic KCl solution (75 mM), and further fixed twice with methanol/acetic (3:1, v/v) mixture, and finally stained on slides with 1× Giemsa solution (G1010, Solarbio, Beijing, China) for 15 min. 200 metaphase cells were analyzed in each group under an optical microscope at 200× magnification (CKX53, Olympus, Tokyo, Japan), respectively, in order to calculate the percentage of chromosomal structural and numerical aberrant cells.

#### 2.8.2. Micronucleus Test

Following the 28-days subacute toxicity test, the pair of femurs was removed from mice to evaluate the micronucleus-inducing potential of CEAE. The proximal epiphyses of femurs were cleaned and sectioned. Then, the bone marrow was removed and washed with saline and further centrifuged at 2000 × g for 10 min. After the supernatant was removed, 2 mL FBS was added to suspend cells. The air-dried smears were fixed with methanol followed by staining with Giemsa solution (G1010, Solarbio, Beijing, China) for 20 min and were finally observed under an oil-immersion lens (1000×). The micronucleated polychromatic erythrocytes (PCEs) were counted per 2000 red cells. The micronucleus-inducing potential was determined by a significant increase in micronucleated PCEs of more than 0.5% when compared with the negative control [18].

### 2.9. Statistical Analysis

The results were expressed as mean ± standard deviation (SD), and the data were analyzed by Tukey's multiple comparison test using GraphPad Prism 8.3.0 (GraphPad Software, San Diego, USA). There was a statistically significant difference between groups when *P* < 0.05.

## 3. Results

### 3.1. Acute Toxicity Test

The mice intragastrically administrated with CEAE at doses of 1000, 2000, 4000, and 5000 mg/kg showed no signs of mortality during the 7 days, indicating that intragastric LD50 value of CEAE in mice was greater than 5000 mg/kg BW. However, 4000 and 5000 mg/kg caused sedation and hyperventilation of mice (Table 1). Additionally, CEAE-treated mice did not manifest significant changes in body weight and relative organ weight in comparison to the control group (*P* > 0.05).

### 3.2. 14-Days Subacute Toxicity Test

#### 3.2.1. Effects of CEAE on the Physiological Indexes for 14 Consecutive Days

Daily intragastric administration of CEAE (400, 800, or 1600 mg/kg) for 14 days did not produce any symptoms of toxicity or mortality but behavioral changes including sedation, hyperventilation, and piloerection were observed at the highest dose level of CEAE (1600 mg/kg) (Table 2). CEAE at doses of 400, 800, and 1600 mg/kg treatment did not also result in significant changes in food intake, water intake, weight gain, and the level of AST, ALT, creatinine, white blood cells, and red blood cells when compared with the control group (*P* > 0.05).

#### 3.2.2. Histopathological Evaluation

**Figure 2:**
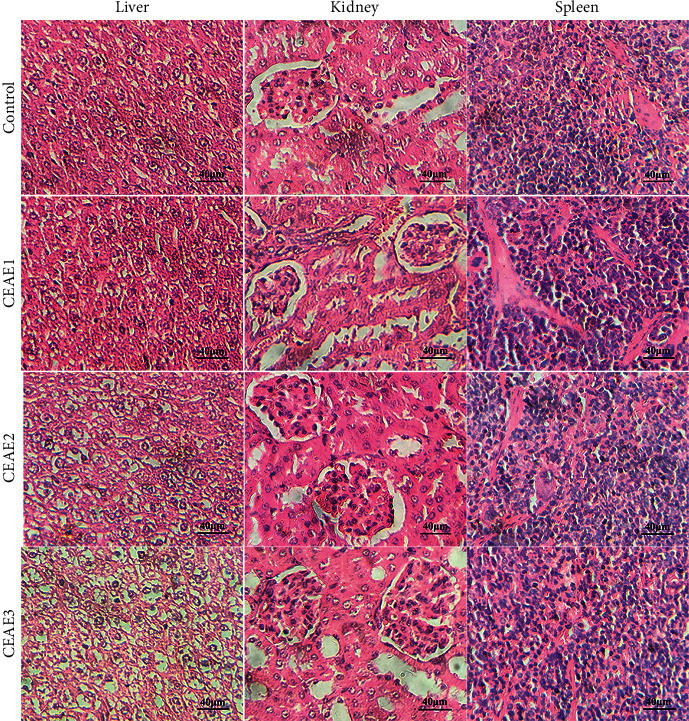
Effects of intragastric administration of CEAE for 14 consecutive days on the liver, kidney, and spleen histomorphology in mice. CEAE: *C. euphlebia* aqueous extract; CEAE1: 400 mg/kg; CEAE2: 800 mg/kg; CEAE3: 1600 mg/kg.

Histological features of the liver, kidney, and spleen of control mice showed normal histomorphology (Figure 2). Administration with 400 mg/kg or 800 mg/kg of CEAE for 14 consecutive days caused no histopathological alterations of the liver in mice. Nevertheless, mice treated with 1600 mg/kg of CEAE for 14 days showed slight hydropic degeneration in the cytoplasm of hepatocytes in comparison to saline-treated mice. Examination of histological sections of the kidney and spleen did not show any remarkable changes or gross pathological changes during the 14-days subacute toxicity test.

### 3.3. 28-Days Subacute Toxicity Test

#### 3.3.1. Effects of CEAE on the Physiological Indexes for 28 Consecutive Days

Daily intragastric administration of CEAE (200, 400, or 600 mg/kg) for 28 days produced no signs of toxicity or mortality (Table 3). CEAE at doses of 200, 400, and 600 mg/kg treatment did not also result in significant changes in food consumption, water consumption, body weight, relative organ weight, creatinine level, number of white blood cells, and the AST and ALT activities, when compared with the control group (*P* > 0.05). Nonetheless, 600 mg/kg of CEAE markedly increased the level of RBC (4.67 ± 0.43 × 10^12^/L) (*P* < 0.05).

#### 3.3.2. Histopathological Evaluation

**Figure 3:**
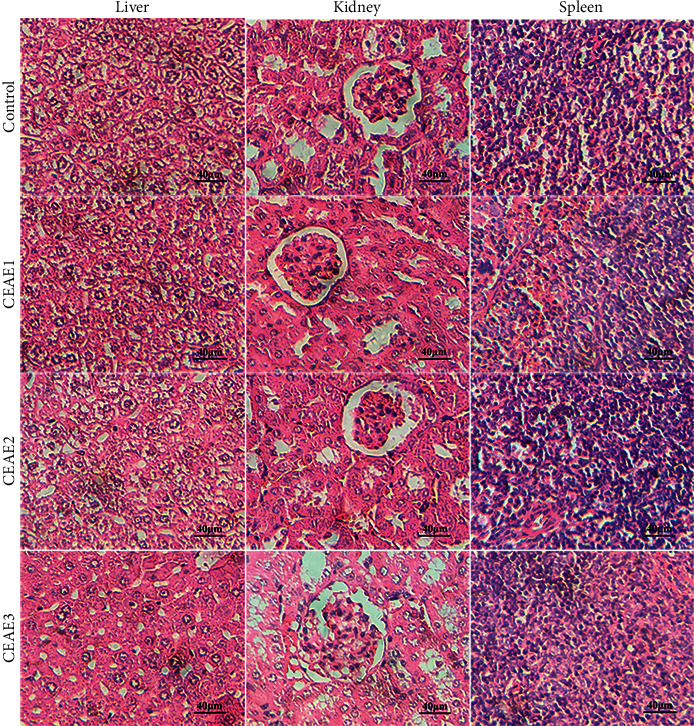
Effects of intragastric administration of CEAE for consecutive 28 days on the liver, kidney, and spleen histomorphology in mice. CEAE: *C. euphlebia* aqueous extract; CEAE1: 200 mg/kg; CEAE2: 400 mg/kg; CEAE3: 600 mg/kg.

Histopathological changes of the liver, kidney, and spleen after intragastric administration of CEAE (200, 400, or 600 mg/kg) for 28 consecutive days in mice were shown in Figure 3. Mice subacute treated with 200, 400, and 600 mg/kg doses of CEAE did not manifest any histopathological alterations of the liver, kidney, and spleen.

### 3.4. In Vitro Chromosomal Aberration Test

**Figure 4:**
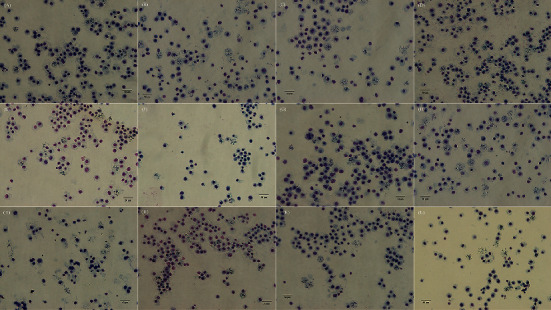
Metaphase CHL cells exposure to various doses of CEAE or positive drugs under an optical microscope at 200× magnification: (a) vehicle control; (b) MMC (0.05 ug/mL) for 6 h treatment without S9 mixture; (c) CPA (10 ug/mL) for 6 h treatment with S9 mixture; (d) CEAE (750 ug/mL) for 6 h treatment without S9 mixture; (e) CEAE (1500 ug/mL) for 6 h treatment without S9 mixture; (f) CEAE (3000 ug/mL) for 6 h treatment without S9 mixture; (g) vehicle control; (h) MMC (0.05 ug/mL) for 24 h treatment without S9 mixture; (i) CPA (10 ug/mL) for 24 h treatment with S9 mixture; (j) CEAE (300 ug/mL) for 24 h treatment with S9 mixture; (k) CEAE (600 ug/mL) for 24 h treatment with S9 mixture; (l) CEAE (1200 ug/mL) for 24 h treatment with S9 mixture.

Both the percentage of chromosomal structural and numerical aberration was 0% in the absence of S9 mixture for 6 h treatment in the vehicle control, whereas the percentage of chromosomal structural and numerical aberration was 0.5% in the presence of S9 mixture for 24 h treatment in the vehicle control (Table 4 and Figure 4). As expected, chromosomal aberration rates induced by MMC and CPA for 6 h without S9 mixture in CHL cells exceeded 14% and even up to more than 30% for 24 h with S9 mixture. However, various doses of CEAE did not significantly increase the percentage of chromosomal aberration including structural and numerical aberrations in all tested conditions (less than 3%), compared with the control, indicating a negative result of CEAE-induced chromosomal aberration in CHL cells.

### 3.5. Micronucleus Test

**Figure 5:**
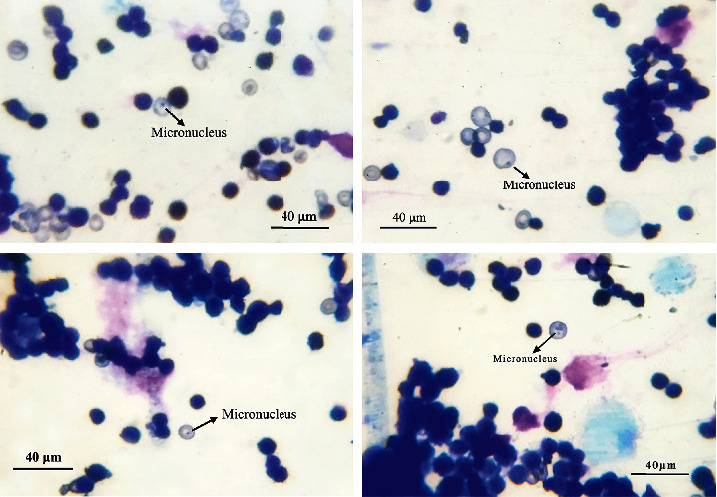
Micrograph of micronucleus in PCEs of murine bone marrow cells. Arrows indicate micronucleated PCEs; calibration bar −40 *µ*m.

Notably, the evaluation of micronucleus-inducing potential showed that the frequency of micronucleated PCEs was less than 0.5% in all groups, and there was no statistically significant difference between the control group (0.18 ± 0.09), CEAE (200 mg/kg, 0.19 ± 0.07%), CEAE (400 mg/kg, 0.27 ± 0.11%), and CEAE (600 mg/kg, 0.20 ± 0.06%) (Table 5 and Figure 5).

## 4. Discussion

The results of our experiments showed for the first time that the aqueous extract of *C. euphlebia* leaves had no potential genotoxicity as demonstrated in the *in vivo* micronucleus test and *in vitro* chromosomal aberration test as well as producing no significant toxic effects except for a transient erythrocytosis and slight hydropic degeneration in the cytoplasm of hepatocytes during acute/subacute toxicity study in mice.

The doses in the acute toxicity study were 1000, 2000, 4000, and 5000 mg/kg, which were equivalent to 60 times, 120 times, 240 times, and 300 times of the effective dose in humans, respectively, according to the conversion algorithm of animal doses to human-equivalent doses based on body surface area [19]. The results of the acute toxicity study indicated that intragastric administration of CEAE at the highest dose of 5000 mg/kg induced no mortality in mice, suggesting an LD50 value upper than 5000 mg/kg (Table 1). Based on the OECD principles [15], orally ingested substances having an LD50 value of more than 5000 mg/kg are relatively safe. Therefore, it can be suggested that CEAE is devoid of acute oral toxicity. In the subacute toxicity study, intragastric administration of CEAE for 14 or 28 consecutive days did not present any alterations in animal symptoms of toxicity or mortality as well as no apparent changes in food intake, water intake, weight gain, and relative organ weight between various groups, but the behavioral changes including sedation, hyperventilation, and piloerection were observed at the highest dose level of CEAE (1600 mg/kg) (Table 2). Combined with other normal physiological indices, these behavioral changes might be common physiological stress symptoms caused by intragastric administration of CEAE.

The liver is the main metabolic center responsible for the biotransformation of xenobiotics in living organisms, and therefore, physiological changes caused by toxic substances may be more obvious in the liver than other organs [20]. The AST and ALT activities in serum and liver tissue are considered to be sensitive markers of hepatotoxicity, and their increased activities indicate different degrees of liver damage (Ozer et al., 2008). Creatinine is recognized as an effective marker of kidney function, and the increase of the creatinine level is associated with renal damage [21]. Blood is the main mobile carrier of substances in the body, and its components are sensitive to toxins and thus, hematological parameters including WBC and RBC represent an important clinical response to toxic compounds [22]. Therefore, in the present study, liver, kidney, and blood functions of all animals were determined after intragastric administration of CEAE during the 14 days or 28 days subacute toxicity experiments. The 14-days subacute toxicity study results showed that CEAE at doses of 400, 800, and 1600 mg/kg treatment did not result in significant changes in the creatinine level, numbers of white blood cells and red blood cells, and the AST and ALT activities in mice; however, mice treated with 1600 mg/kg of CEAE appeared slight hydropic degeneration in the cytoplasm of hepatocytes (Table 2 and Figure 2). The inconsistent results between histopathological and biochemical evaluation were explicable, with the likelihood that slight hydropic degeneration in the cytoplasm of hepatocytes did not cause abnormal activities of ALT and AST in the liver tissue. In the 28-days subacute toxicity study, CEAE at low, medium, and high doses did not result in significant changes in food intake, water intake, body weight, relative organ weight, AST and ALT activities, creatinine level, number of white blood cells, and morphology of the liver, kidney, and spleen compared with the control group (Table 3 and Figure 3). Surprisingly, 600 mg/kg of CEAE significantly increased the number of RBC. Given no gross morphological, pathological, and physiological abnormalities of the liver and kidney, the erythrocytosis caused by the administration of CEAE is less likely to be related to an increase in erythropoietin, a well-known hematopoietic factor produced by the kidney and liver to stimulate erythropoiesis [23]. The erythrocytosis was more inclined to be transient, and further *in vivo* or *in vitro* study involved in erythropoietin testing or kidney function should be performed to understand the reason behind it.

The evaluation of micronucleus-inducing potential using the micronucleus test is recommended by the US Food and Drug Administration and frequently used for evaluating the genotoxic risk of various types of substances [24, 25]. The results showed that the incidence of micronucleated PCEs in all mice was less than 0.5% within the normal range [26] and no statistically significant difference between various groups, demonstrating that CEAE had no micronucleus-inducing potential and no harmful effect on cell mitosis of PCEs (Table 5). Only the micronucleus test result was unable to obtain accurate genotoxicity (mutagenicity) information; therefore, further genotoxicity test is required for confirmation of genotoxicity of CEAE. In addition to the micronucleus test, other *in vitro* assay systems have been also used to evaluate the genotoxic potential of tested substances including the Ames test, chromosomal aberration test, and Comet assay [27–29]. In this study, the *in vitro* chromosomal aberration test in CHL cells indicated that CEAE did not produce chromosomal damage-inducing potential (Table 4), further underpinning the ascertainment of nongenotoxicity of CEAE. Last but not least, CEAE was not a single-sex antidepressant drug; so, single-sex animals were not chosen as tested subjects in this study. Moreover, it is particularly explained that the experimental design (equal male-to-female ratio in each group) effectively evaluate the toxicity of CEAE in animals, albeit animals of different sexes possibly appear a different sensitivity to the same drug [30, 31].

## 5. Conclusions

In the present toxicological study, CEAE caused slight hydropic degeneration in the cytoplasm of hepatocytes and transient erythrocytosis, whereas showing no potential genotoxicity and adverse effects on other physical, biochemical, and hematological parameters in animals. These preliminary experimental data demonstrated its low toxicity in mice. Further chronic toxicological study focusing on hepatotoxicity and erythrocytosis should be carried out systematically to support the safety use of CEAE for functional food or therapeutic drugs (especially for antidepressant agents) and protect the subjects from potential toxic effects of the extract.

## Figures and Tables

**Table 1:** Effects of intragastric administration of CEAE three times a day on mice.

Parameters	Groups^*∗*^				
	Control	CEAE (1000 mg/kg)	CEAE (2000 mg/kg)	CEAE (4000 mg/kg)	CEAE (5000 mg/kg)
Mortality (%)	0	0	0	0	0
Toxic symptoms	None	None	None	Sedation and hyperventilation	Sedation and hyperventilation
Body weight (g)	30.33 ± 2.65^a^	29.90 ± 1.51^a^	30.35 ± 1.55^a^	30.08 ± 2.55^a^	31.08 ± 1.61^a^
Liver weight (%)	2.32 ± 0.08^a^	1.98 ± 0.23^a^	2.15 ± 0.48^a^	2.29 ± 0.23^a^	2.27 ± 0.23^a^
Kidney weight (%)	0.53 ± 0.04^a^	0.47 ± 0.06^a^	0.50 ± 0.09^a^	0.44 ± 0.03^a^	0.50 ± 0.08^a^
Spleen weight (%)	0.13 ± 0.01^a^	0.14 ± 0.01^a^	0.15 ± 0.02^a^	0.14 ± 0.04^a^	0.14 ± 0.03^a^

^*∗*^Within each row, values with the same superscripts are not significantly different from each other (*P* < 0.05), as determined by Tukey's *t*-test. CEAE: *C. euphlebia* aqueous extract.

**Table 2:** Effects of intragastric administration of CEAE for consecutive 14 days on the physiological indices in mice.

Parameters	Groups^*∗*^			
	Control	CEAE (400 mg/kg)	CEAE (800 mg/kg)	CEAE (1600 mg/kg)
Mortality (%)	0	0	0	0
Toxic symptoms	None	None	None	Sedation, piloerection, and hyperventilation
Food intake (g)	7.6	9.2	8.4	7.6
Water intake (mL)	12	8.0	7.0	12
Weight gain (g)	4.16 ± 0.64^a^	4.18 ± 0.73^a^	3.43 ± 0.99^a^	3.32 ± 1.12^a^
Liver weight (%)	4.32 ± 0.44^a^	4.40 ± 0.87^a^	4.30 ± 0.36^a^	4.48 ± 0.35^a^
Spleen weight (%)	0.31 ± 0.04^a^	0.32 ± 0.04^a^	0.31 ± 0.04^a^	0.34 ± 0.04^a^
Kidney weight (%)	1.20 ± 0.12^a^	1.27 ± 0.10^a^	1.20 ± 0.10^a^	1.23 ± 0.11^a^
White blood cells (×10^9^/L)	5.95 ± 0.78^a^	5.41 ± 0.59^a^	5.83 ± 1.01^a^	5.69 ± 1.15^a^
Red blood cells (×10^12^/L)	6.46 ± 1.24^a^	5.57 ± 1.27^a^	5.39 ± 0.98^a^	5.36 ± 1.07^a^
Creatinine (*µ*mol/L)	62.62 ± 7.48^a^	59.56 ± 8.55^a^	61.50 ± 6.90^a^	59.66 ± 10.70^a^
AST (U/gprot)	65.99 ± 7.18^a^	59.12 ± 7.26^a^	65.53 ± 8.13^a^	66.15 ± 8.61^a^
ALT (U/gprot)	23.93 ± 4.84^a^	26.92 ± 4.95^a^	30.19 ± 5.52^a^	30.79 ± 8.14^a^

^*∗*^Within each row, values with the same superscripts are not significantly different from each other (*P* < 0.05), as determined by Tukey's *t*-test. CEAE: *C. euphlebia* aqueous extract.

**Table 3:** Effects of intragastric administration of CEAE for consecutive 28 days on the physiological indices in mice.

Parameters	Groups^*∗*^			
	Control	CEAE (200 mg/kg)	CEAE (400 mg/kg)	CEAE (600 mg/kg)
Mortality (%)	0	0	0	0
Toxic symptoms	None	None	None	None
Food intake (g)	10.30	8.95	9.70	9.80
Water intake (mL)	14	13	12	13
Body weight (g)	39.74 ± 2.05^a^	39.07 ± 2.48^a^	39.89 ± 3.52^a^	40.95 ± 2.20^a^
Liver weight (%)	4.96 ± 0.05^a^	4.95 ± 0.43^a^	4.89 ± 0.26^a^	4.78 ± 0.42^a^
Spleen weight (%)	0.30 ± 0.06^a^	0.38 ± 0.08^a^	0.22 ± 0.07^a^	0.35 ± 0.14^a^
Kidney weight (%)	1.33 ± 0.12^a^	1.41 ± 0.03^a^	1.50 ± 0.15^a^	1.57 ± 0.21^a^
White blood cells (×10^9^/L)	3.44 ± 0.46^a^	3.70 ± 0.35^a^	3.59 ± 0.50^a^	3.85 ± 0.46^a^
Red blood cells (×10^12^/L)	3.48 ± 0.57^a^	4.02 ± 0.78 ^ab^	4.79 ± 0.72 ^ab^	4.69 ± 0.43^b^
Creatinine (*µ*mol/L)	52.34 ± 7.31^a^	59.09 ± 8.53^a^	55.03 ± 9.72^a^	65.37 ± 10.70^a^
AST (U/L)	129.34 ± 12.73^a^	131.26 ± 22.57^a^	140.97 ± 21.31^a^	145.26 ± 17.91^a^
ALT (U/L)	49.88 ± 11.27^a^	54.94 ± 15.31^a^	56.42 ± 14.35^a^	64.34 ± 10.88^a^

^*∗*^Within each row, values with different superscripts are significantly different from each other (*P* < 0.05), as determined by Tukey's *t*-test. CEAE: *C. euphlebia* aqueous extract.

**Table 4:** *In vitro* chromosomal aberration test of CEAE in CHL fibroblast cells in the absence or presence of S9 metabolic activation.

Drugs^*∗*^	Doses (ug/mL)	Treatment time (h)	Recovery time (h)	S9 mixture	Structural aberrations (%)	Numerical aberrations (%)
Vehicle control	−	6	18	−	0	0
Mitomycin C	0.05	6	18	−	14.5	0.5
Cyclophosphamide	10	6	18	+	19.5	1.5
CEAE	750	6	18	−	0.5	0
CEAE	1500	6	18	−	2.5	1.5
CEAE	3000	6	18	−	1.5	0
Vehicle control	−	24	0	+	0.5	0.5
Mitomycin C	0.05	24	0	−	31	1
Cyclophosphamide	10	24	0	+	43.5	2.5
CEAE	300	24	0	+	0.5	0
CEAE	600	24	0	+	1	0
CEAE	1200	24	0	+	1	0.5

^*∗*^CEAE: *C. euphlebia* aqueous extract.

**Table 5:** Effects of intragastric administration of CEAE for consecutive 28 days on the micronucleus rate in PCEs of murine bone marrow cells.

Drugs	Dosage (mg/kg)	Total number of micronucleus	Micronucleus frequency (%)^*∗*^
Control	—	21	0.18 ± 0.09^a^
CEAE	200	23	0.19 ± 0.07^a^
CEAE	400	32	0.27 ± 0.11^a^
CEAE	600	24	0.20 ± 0.06^a^

^*∗*^Within each row, values with different superscripts are significantly different from each other (*PP* < 0.05), as determined by Tukey's *t*-test. CEAE: *C. euphlebia* aqueous extract.

## Data Availability

The data used to support the findings of this study are available from the corresponding author upon request.
